# Semi-Annual Meeting Erie County Medical Society

**Published:** 1864-06

**Authors:** 


### SEMI-ANNUAL MEETING ERIE COUNTY MEDICAL SOCIETY.

The Semi-Annual Meeting of this Society was held June 14th, and was well attended. The exercises in the morning consisted in admission of new members and the transaction of miscellaneous business. The afternoon session was assigned for hearing the Address. Dr. Dorland of Hamburgh, was the regularly appointed orator. We were not able to attend the afternoon meeting and cannot speak of the proceedings.

The following gentlemen were elected to membership on compliance with the by-laws:

| | |
|---|---|
| Dr. H. Van Guysling, | Buffalo. |
| " E. B. Tefft, | " |
| " J. C. Greene, | " |
| " A. J. Houghton, | Tonawanda. |
| " E. B. Horton, | Eden. |
| " J. S. Havens, | Aurora. |
| " O. W. Beckwith, | Evans. |
| " U. C. Lynde, | Springville. |
| " P. Goodyear, | Alden. |

The Secretary was directed to call the attention of members to the law requiring a copy of the diploma to be filed in the County Clerk's Office before membership can be completed.

